# Common Virulence Factors and Tissue Targets of Entomopathogenic Bacteria for Biological Control of Lepidopteran Pests

**DOI:** 10.3390/insects5010139

**Published:** 2014-01-06

**Authors:** Anaïs Castagnola, S. Patricia Stock

**Affiliations:** 1 Center for Insect Science, University of Arizona, 1007 E. Lowell Street, Tucson, AZ 85721, USA; E-Mail: anais@email.arizona.edu; 2 Department of Entomology, University of Arizona, 1140 E. South Campus Dr., Tucson, AZ 85721, USA

**Keywords:** *Photorhabdus*, *Bacillus thuringiensis*, virulence factors, toxins, neurobiology, Mcf, Tc, Cry

## Abstract

This review focuses on common insecticidal virulence factors from entomopathogenic bacteria with special emphasis on two insect pathogenic bacteria *Photorhabdus* (Proteobacteria: Enterobacteriaceae) and *Bacillus* (Firmicutes: Bacillaceae). Insect pathogenic bacteria of diverse taxonomic groups and phylogenetic origin have been shown to have striking similarities in the virulence factors they produce. It has been suggested that the detection of phage elements surrounding toxin genes, horizontal and lateral gene transfer events, and plasmid shuffling occurrences may be some of the reasons that virulence factor genes have so many analogs throughout the bacterial kingdom. Comparison of virulence factors of *Photorhabdus*, and *Bacillus*, two bacteria with dissimilar life styles opens the possibility of re-examining newly discovered toxins for novel tissue targets. For example, nematodes residing in the hemolymph may release bacteria with virulence factors targeting neurons or neuromuscular junctions. The first section of this review focuses on toxins and their context in agriculture. The second describes the mode of action of toxins from common entomopathogens and the third draws comparisons between Gram positive and Gram negative bacteria. The fourth section reviews the implications of the nervous system in biocontrol.

## 1. Introduction

For many decades, naturally occurring microbial insect pathogens, including bacteria, fungi, microsporidia, protists, viruses, and nematodes have been considered as non-chemical alternatives for suppression of insect pests [1,2,3]. Positive attributes such as their high specificity and less damage to non-target fauna and flora has certainly contributed to the consideration in management practices of agricultural and forest insect pests [4].

Of all entomopathogens, bacteria have been the most extensively used organisms to date. Indeed, *Bacillus thuringiensis* (Bt) (Firmicutes: Bacillaceae), originally discovered in 1901 by Ishiwata [5] and later rediscovered by Berliner [6], has been the most studied and broadly used in microbial control. In particular, the discovery that Bt spore-associated toxins are extremely virulent and can persist in the environment with high potency, prompted the development of bacterial spray formulations [7] and transgenic plants expressing bacterial toxins [8,9]. Advancements in the characterization of bacterial pathogens including purification and culturing methods, molecular identification of virulence factors, and whole genome characterization and comparisons have prompted the discovery of novel pest management tools. In this vein, insecticidal molecules expressed and secreted by various entomopathogenic bacteria have been targeted for genetic manipulation to enhance toxicity [10,11].

Recently, other insect pathogenic bacteria with modes of action similar to Bt have been hailed as agriculturally relevant [12]. Certainly, insect pathogenic bacteria of diverse taxonomic groups and phylogenetic origin have been shown to have striking similarities in the virulence factors they produce. [13]. Bacterial virulence factors are often encoded on mobile genetic elements, such as plasmids and bacteriophages, and can easily be spread through horizontal gene transfer. The discovery that both Gram-negative and Gram-positive bacteria produce analogous insect-specific toxins infers history of gene transfer between these organisms [14]. For example, *Photorhabdus luminescens* (Proteobacteria: Enterobacteriaceae), the bacterial symbiont of the entomopathogenic nematode *Heterorhabditis bacteriophora* (Rhabditida: Heterorhabditidae), has been shown to have similar virulence factors to that of Bt [15].

Herein, we review the commonalities between virulence factors produced by entomopathogenic bacteria. Special emphasis is placed on those with potential for control of lepidopteran pests. Additionally, we discuss and propose novel tissue targets of virulence factors for their potential application in pest management.

## 2. Toxins Produced by Entomopathogenic Bacteria

Insecticidal toxins used in agriculture are predominantly from Gram-positive bacteria and derived mostly from *Bacillus thuringiensis*. Foliar sprays containing *B. thuringiensis* represent an organic alternative to synthetic foliar sprays. Transgenic plants expressing Cry toxins are now viewed as an overwhelming ecological success [16].

Entomopathogenic Gram-negative bacteria also produce toxins. Based on their targeted tissues, they can be categorized into three types (a) neurotoxins; (b) digestive toxins; and (c) cytotoxins. Members in the Enterobacteriaceae such as *Photorhabdus*, *Xenorhabdus*, *Serratia*, and *Yersinia* spp. produce insecticidal toxins with oral toxicity similar to that of Bt toxins, but have not yet been fully utilized.

This section describes the virulence factors associated with both Gram-negative and Gram-positive bacteria as well as their mode of action.

### 2.1. Insecticidal Toxin Genes Produced by Gram Positive Bacteria

#### 2.1.1. Bacillaceae

*Bacillus* spp.: Within this genus three species, *B. thuringiensis*, *Bacillus cereus*, *Bacillus anthracis* are the most widely studied taxa in terms of insecticidal toxins. Recent phylogenetic studies based on 16S and 23S rRNA sequence data consider *B. thuringiensis*, together with *B. cereus*, *B. anthracis* and *Bacillus mycoides* (Firmicutes: Bacillaceae) nearly identical. However, their unique pathogenicity properties and the diverse modes of actions of their insecticidal toxins (Table 1) support their distinctiveness, giving each separate species names [13].

*Bacillus thuringiensis* is a phylloplane and soil-dwelling bacterium that is aerobic and endospore-forming. It has been isolated from many insect carcasses and a few plants, where it has been reported as an endophyte. Upon sporulation, *B. thuringiensis* forms crystals of proteinaceous insecticidal δ-endotoxins, called crystal proteins or Cry proteins, which are encoded by *cry* genes [17]. The Cry toxins, are insecticidal to many insect orders including Lepidoptera, Diptera [18,19], Coleoptera [20], and Hymenoptera [21], and also have activity against nematodes [22,23]. Upon ingestion by Lepidoptera larvae, Cry protoxins are first solubilized by the alkaline pH of the gut, then proteolytically activated by proteases. The insecticidal form of these toxins has been shown to bind to specific receptors on the columnar cells of the midgut epithelium causing pore formation and midgut cell death. Cry toxins are commonly classified as gut poisons, as they compromise the epithelial-hemocoel layer and ultimately lead to starvation and/or septicemia and larval death [24]. Cry toxins bind, form pores, and damage midgut epithelial goblet cells in mixed midgut cell cultures [25] and *in vivo* [26] and have also been shown to bind to the insect’s peritrophic matrix [27]. The Cry toxins from *B. thuringiensis* are almost exclusively considered digestive toxins; however, they have homology to neurotoxins and effect diverse tissues of lepidopteran larvae. They have been shown to kill larval neurons of the cerebral ganglia *in vitro* [28], invade liposomes causing morphological changes to lipid bilayers *in vitro* [29], initiate apoptosis-like pathway in ovary-derived cells expressing a Bt receptor [30], and bind ATP-binding cassette (ABC) transporters [31]. In this respect, mutated ABC transporters are correlated with resistance to Bt toxins in domesticated silkworm, *Bombyx mori* (Lepidoptera: Bombycidae) [32], tobacco budworm, *Heliothis virescens* (Lepidoptera: Noctuidae) [33], diamond back moth, *Plutella xylostella* (Lepidoptera: Plutellidae), and cabbage looper, *Trichoplusia ni* (Lepidoptera: Noctuidae) [34].

**Table 1:** Mode of action of entomopathogenic bacterial toxins with toxicity to lepidopteran larvae.

Family	Species	Description	Name
Gram Positive			
Bacillaceae	*B. thuringiensis*	Crystal toxins produced during sporulation, pore forming toxins that act on gut columnar cells	Cry toxins
		Pore forming toxin that acts on gut columnar cells, causes death to insect brains cells	Cry1C toxin
		Vegetative insecticidal protein produced during the vegetative growth cycle, binds to midgut receptors, gut paralysis, lysis of midgut columnar cells	Vip3 toxin
		Associated with the presence of certain *cry* and *vip* genes, toxic to mammalian cells, growth inhibition, secreted during vegetative growth cycle	Β-exotoxin I
	*B. thuringiensis israelensis*	TccC and TcaCBA toxin homologs to *Photorhabdus* and *Yersinia*	Strain bthur0013
	*B. subtilis*	Biosurfactant, destructive effect on gut epithelial cells by vesicle formation in the apical region and cellular vacuolization	SPB1
	*B. cereus*	Secreted non-proteinous exotoxins, pathogenic to humans and insects	Exotoxin
	*L. sphaeriscus*	Cholesterol binding cytolysin, lyses neurons and hemocytes	Sphaericolysin
Clostridiaceae	*C. botulinum*	Binary actin binding-ADP-ribosylating exotoxin	C2
	*C. difficile*	Exotoxin which reorganizes cytoskeleton, hydrophobic central domain is shared between the Mcf1 and Mcf2 toxins, cytotoxic motif	Cytotoxin B
Gram negative			
Enterobacteriaceae	*S. entomophila*	Shows high similarity to *P. luminescens* TcbA, TcdA, TcaB, and TccB, tripeptide cell-binding motif Arg-Gly-Asp implicates the ability to bind to eukaryotic cells	SepA
		Shows similarity to *P. luminescens* TcaC and SpvB	SepB
		Shows similarity to *P. luminescens* TccC, Shows similarity to *Bacillus subtilis* WapA, *E. coli* Rhs, *Streptomyces coelicolor* A3, *Coxiella burnetii*	SepC
		Antifeeding prophage causes cessation of feeding	Afp
	*Y. frederiksenii*	Orally toxic, not subcutaneously toxic, high homology to *sep* genes in *Serratia*	TcYF1 and TcTF2
	*P. luminescens*	Assists TcC to translocate into the cell, transmembrane pore formation, contains an integrin binding motif	TcA (TcdA1)
		Potentiates expressed toxin genes *tcdA1*, *tcaA*, and *tcaB*	TcdB1, TccC1, TcaC, TcdB2, TccC3
		Assists TcC (TccC3) to translocate in the cell	TcB (TcdB2)
		Actin-clustering, defects in phagocytosis and cell death	TcC (TccC3)
		Formation of actin aggregates, ADP-ribosylates actin at the threonine-148 with TccC3 sub-complex	Tc Complex
		Makes caterpillars floppy phenotype, induce apoptosis, rearrange actin	Mcf
		Degrades peritrophic matrix, causes midgut damage, midgut cell sloughing, fat body nuclear degradation	Txp40
		*Photorhabdus* virulence cassettes, phage-like, kills and condenses actin of hemocytes	PVC
		Primarily hemoplymph-based insecticidal activity	pirA2B2, locus plu4437-plu4436
		Similar to *Serratia*-type hemolysins	phlBA operon
		Binds actin ADP-ribosylating, inhibits actin polymerization	Photox
	*X. nematophila*	Midgut, intestinal sloughing	A24tox
		Fed to neonates and caused inhibitory growth	Xin
		From the cosmid CHRIM1, analogs to *sep Serratia* genes, oral toxicity	XptA1, XptA2, XptB1, XptC1
		Alphaxenorhabdolysin triggers apoptosis in hemocyte cells, cytotoxic and hemolysin effects, has analogs in *Photorhabdus*, *P. entomophila*, *P. syringae*, *Y. enterocolitica*, and *Proteus mirabilis*	xaxAB
		High similarity to GroEL, injectable toxicity to *G. mellonella*, innate immune response of increased phenoloxidase activity stimulated by injection	HIP57
		One gene in the xenocin operon with RNAse and cytotoxic activity	xciA
Pseudomonadaceae	*P. fluorescens*	Related to the Mcf from *P. luminescens*, hemolymph-based insecticidal activity	Fit
		Regulators of Fit insect toxin expression for biocontrol	FitG
	*P. entomophila*	TccC-type toxins	PSEEN2485, PSEEN2697, PSEEN2788
		TcdB-type toxin	PSEEN1172
		Tcc-C type toxins	PSEEN701, PSEEN702
		Exotoxins with hemolytic activity	PSEEN3925, PSEEN0968, PSEEN3843
		Lipases	PSEEN709, PSEEN1065, PSEEN2195, PSEEN3432
	*P. syringae*	Tcc-C type toxins	PSEEN701, PSEEN702
		TcdB-type toxin	PSEEN1172

*Bacillus cereus* has been mostly studied for its role in food contamination and human digestive food poisoning; however, this bacterium associates with soil and plants much like Bt [35]. As mentioned above, *B. cereus* and *B. thuringiensis* are two very closely related taxa, however they produce different types of toxins. Unlike Bt, the endospore of *B. cereus* is not insecticidal. Both *B. thuringiensis* and *B. cereus* produce non-proteinous insecticidal exotoxins during their vegetative growth cycle. β-Exotoxin I is produced by *B. thuringiensis* and a small, proteinous exotoxin is produced by *B. cereus* [36]. Although, *B. cereus* has been shown to grow and proliferate in the insect gut, this bacterium is mostly regarded as an opportunistic pathogen with the production of virulence factors that are most effective when titers are high. Entomopathogenic toxin genes have also been found in the genome of strain *B. thuringiensis israelensis* bthur0013, a member of the *B. cereus* sensu lato [37].

Other *Bacillus* species known to produce insecticidal components are *Bacillus circulans* (Firmicutes: Bacillaceae) and *sphaeriscus* (Firmicutes: Bacillaceae) (=*Lysinibacillus sphaeriscus*). Virulence factors produced by the former sp. have been shown to affect insects (mostly dipterans) and other invertebrates such as nematodes, and mollusks [38]. Sphaericolysin is a toxin produced during the vegetative growth cycle of *L. sphaeriscus* [39]. This toxin has heamoceolic toxicity toward *Blattela germanica* (Blattodea: Blattellidae) and *Spodoptera litura* (Lepidoptera: Noctuidae) [40].

#### 2.1.2. Clostridiaceae

*Clostridium* spp.: *Clostridium* are anaerobic spore-forming bacteria and, similar to *Bacillus*, comprise over one hundred species. Similar to *Bacillus*, *Clostridium* spp. produce binary proteinous toxins that are proteolytically activated by serine proteases [41] (Table 1). For example, *Clostridium bifermentans* serovar *malaysia* produces larvacidal toxin proteins that are active against mosquitoes [42]. *Clostridium perfringens* (Firmicutes: Clostridiaceae) has an iota toxin which binds actin by ADP-ribosylation and has a C-domain structure similar to that of the vegetative insecticidal protein of Bt, VIP2, although this protein ultimately targets mammals [43]. The *Clostridium difficile* cytotoxin B is an exotoxin which causes the reorganization of cytoskeletons, similar to Mcf (Makes Caterpillars Floppy) toxins produced by Gram-negative *P. luminescens* (See Section 2.2). Moreover, they have a three domain structure consisting of receptor-binding, translocation, and one catalytic domain [44]. 

The actin binding C2 toxin of another *Clostridium* sp., *C. botulinum*, is a binary toxin with four domains of activation, pore formation, and receptor recognition. The C2 toxin is similar to the toxin complex, TccC3, produced by *P. luminescens* because they both have ADP-ribosylating activity and bind actin. One difference between these two toxins is that TccC3 forms a complex with other Tc toxins, TcdA1 and TccC3, and is therefore not binary. Although ADP-ribosylation occurs in both cases, TccC3 ribosylates at a threonine while C2 toxins ribosylate at arginine, dysfunctioning actin by inducing actin polymerization in the former and inhibition in the latter [45]. A toxin found in *P. luminescens* has an identical mode of action to that of C2 toxin. The translated product of the *plu0822* gene, termed Photox toxin, stops actin polymerization by targeting an arginine with ADP-ribotransferase activity [46]. 

### 2.2. Insecticidal Toxin Genes Produced by Gram Negative Bacteria

#### 2.2.1. Enterorbacteriaceae

*Photorhabdus* spp.: Until now three species have been identified within this genus*: P. luminescens*, *Photorhabdus temperata* and *Photorhabdus asymbiotica* [47]. All *Photorhabdus* species are mutualistically associated with insect parasitic *Heterorhabditis* nematodes. However, *P. asymbiotica* has also been found associated with skin injuries in human patients [48]. This bacterium is currently considered an emerging human pathogen model system [49]. Unlike *B. thuringiensis*, *Photorhabdus* spp. are facultative anaerobes and cannot live freely in the soil environment. These bacteria are vectored by the nematodes and together they form an insecticidal complex that kills the insect and use the carcass for reproduction and nutrition. Once delivered by the nematodes in the insect hemocoel, the bacteria first evade the insect immune system and then produce toxins which kill the insect and break down insect epithelial tissues. The *Photorhabdus* genome contains a multitude of pathogenicity islands with an abundance of toxin genes [50]. The major virulence factors characterized so far consist of the *mcf1 and mcf2* (makes caterpillars floppy) genes, the *tc* (toxin complex) genes, Pir (*Photorhabdus* insect related) operon, and a multitude of virulence factors associated with the *Photorhabdus* virulence cassettes (PVC) (Table 1) [51].

The Mcf toxins are known to both rearrange actin cytoskeletons and induce apoptosis in both hemocytes and epithelial tissue, resulting in an abundance of tissue damage. The intensity of the tissue damage is so great that there is a complete loss of turgor pressure throughout the infected insect [52].

The Tc proteins, similar to Bt Cry toxins, are orally toxic compounds that have been discovered to be insecticidal to a wide range of insect taxa including Coleoptera, Lepidoptera, and Hemiptera. It has been shown that *P. luminescens* is highly pathogenic when injected into lepidopteran hosts such as the African cotton leafworm, *Spodoptera littoralis* (Lepidoptera: Noctuidae), and *P. xylostella*. The oral toxicity of the Tc proteins is peculiar because *Photorhabdus* are delivered directly into the hemocoel of the target insect. Furthermore, the Tc proteins are active in the lumen side of the insect’s midgut epithelium and not in the basal side of this tissue, which would be the expectation for a hemocoel-initiating pathogen [53].

Pir proteins are other *Photorhabdus* toxins that have been identified to have hemolymph [54] and oral [55] toxicity. The Pir proteins have homology to a neurotoxin commonly called leptinotarsin [55,56,57] and are binary [51]. The binding and destructive effects on neural tissue could be a major factor in toxicity when *Photorhabdus* are injected into susceptible insects.

It can be speculated that there are many more toxins yet to be characterized from the *Photorhabdus* genome which are responsible for hemolymph-based toxicity. The Txp40 protein has been identified in 59 different strains of both *Photorhabdus* and *Xenorhabdus*. It has injectable toxicity to many lepidopteran insects including the greater wax moth, *Galleria mellonella* (Lepidoptera: Pyralidae), Indian meal moth, *Plodia interpunctella* (Lepidoptera: Pyralidae), corn earworm, *Helicoverpa armigera* (Lepidoptera: Noctuidae), and the Dipterans such as the Australian sheep blowfly, *Lucilia cuprina* (Diptera: Calliphoridae) [58]. A *txp40* gene, when expressed by *E. coli*, has also been shown to be insecticidal to *P. xylostella* [59]. The Txp40 protein is involved in causing damage to both the insect midgut and the fat body and has also been shown to exhibit cytotoxicity *in vitro* in dipteral and lepidopteran cell lines [58].

*Xenorhabdus* spp.: Similar to *Photorhabdus*, bacteria in this genus are also non-free-living, though they are symbiotically associated with nematodes in the genus *Steinernema* [60]. Similar to *Heterorhabditis*, *Steinernema* nematodes also play a key role in vectoring these bacteria from one insect host to another. *Xenorhabdus* also produce a large number of insecticidal toxins to help them garner nutrients from the insect hosts (Table 1). One example is a toxin from *Xenorhabdus nematophila* (Proteobacteria: Enterobacteriaceae) called A24tox. A24tox is a 42 kD protein that has been shown to kill *G. mellonella* and *H. armigera*. This toxin has a hypothetical homolog in *Photorhabdus* but has no significant matches outside of this group [61]. Another example is the xenocin operon. The xenocin operon consists of two genes, *xciA* and *ximB*. When expressed, these molecules are secreted by the flagellar type II secretion pathway. Xenocin, the *xciA* gene, has RNAse activity and cytotoxicity. The immunity protein co-expressed in the xenocin operon has antimicrobial effects killing competing microbes in insect larvae [62].

The *xpt* gene products from *X. nematophila* have oral insecticidal activity to different lepidopteran larvae based upon how the differentially expressed *xpt* genes interact with each other. In this respect, the translated products of Tc genes are similar to the Tc proteins from *Photorhabdus*. The Tc gene products can be categorized into toxins and potentiators. Potentiators synergize with their Tc toxin counterpart for full insecticidal activity [53]. When *xptA1*, *xptA2*, *xptB1*, and *xptC1* genes are expressed in *Escherichia coli* and are fed to larvae, they confer oral insecticidal toxicity to various lepidopteran spp. including: cabbage butterfly, *Pieris brassicae* (Lepidoptera: Pieridae), white butterfly, *Pieris* (Lepidoptera: Pieridae), and *H. virescens*. Interestingly, when the interactions of all four genes are separated from each other their insecticidal toxicity is different. For example, *E. coli* expressed *xptA1*, *xpbtB1*, and *xptC1* are needed to be insecticidal toward *P. rapae* and *P. brassicae*. The addition of *xptA2* and the removal of *xptA1* is all that is needed for insecticidal toxicity toward *H. virescens*. Thus, different combinations of the *xpt E. coli* lysates show different insecticidal specificities to different lepidopteran larvae [63]. The intracellular Xin toxic protein from *X. nematophila* has also been demonstrated to have an inhibitory growth effect on cotton bollworm, *Helicoverpa* (Lepidoptera: Noctuidae), after ingestion with artificial diet incorporating the Xin protein [64].

The *xaxAB* genes from *X. nematophila* expressed in *E. coli* induce apoptosis when incubated with hemocytes derived from *S. littoralis*. Additionally, the order in which Xax toxins (XaxA and XaxB) are added to cells *in vitro* affects their toxicity [65]. This is similar to *xpt* and *tc* genes that are expressed in *E. coli*.

*Xenorhabdus* bacteria also produce an insecticidal protein called HIP57 that have high similarity to chaperonins like GroEL produced by *E. coli*. GroEL chaperonins have been shown to help proteins fold from non-native to native structures [66]. Moreover, they help combat problems such as aggregation when nascent proteins have hydrophobic residues exposed before reaching a fully folded native state [67]. HIP57 has been shown to have injectable toxicity to *G. mellonella*. The insecticidal property of HIP57 is a novel function for the GroEL superfamily of proteins. Moreover, an innate immune response of increased prophenoloxidase activity is stimulated by HIP57 injection [68].

*Serratia* spp.: These bacteria often exist as endophytes possessing fungicidal properties, but can also associate with insects [69] and nematodes [70,71] in a facultative manner. Genome studies have found several insecticidal genes in the *Serratia* genome (Table 1). Other *Serratia* sp. are responsiblefor causing amber disease in grass grubs, *Costelytra zealandica* (Coleoptera: Scarabaeidae) [72]. Contrastingly, *Serratia marcescens* (Proteobacteria: Enterobacteriaceae) infects other host such as poorly reared *H. virescens* [73]. The pADAP plasmid from *Serratia entomophila* contains the genes *sepA*, *sepB*, and *sepC*. These genes are similar to the Tc genes described in *P. luminescens* and the *xpt* genes identified from *X. nematophila.* There is no need for the entire pADAP plasmid to be associated with the *sep* genes for amber disease to occur and cause death. However, when the *sep* genes are expressed in *E. coli* without the entire plasmid, the scarab beetles do not cease feeding [74] which is one symptom of amber disease. The virulence factor associated with pADAP that causes cessation of feeding in amber disease is the anti-feeding prophage (Afp) [75]. Therefore both the *sep* genes and Afp are needed for full virulence of *Serratia* in grass grubs.

*Yersinia* spp.: Members of this genus are facultative anaerobes. In particular, *Yersinia pestis* the causative agent of bubonic disease (aka plague) is associated with fleas, humans and rodent intermediates. Two other species which are pathogenic to humans are *Yersinia enterocolitica* and *Yersinia pseudotuberculosis* often causing diarrheal disease and fever with inflammation, respectively. *Yersinia entomophaga* (Proteobacteria: Enterobacteriaceae), *Yersinia frederiksenii* [76], which can cause disease in grass grubs, *Y. enterocolitica* [77], *Y. pseudotuberculosis*, *Yersinia mollaretii* (Proteobacteria: Enterobacteriaceae) [78] and *Y. pestis* [79] have insecticidal genes of the Tc super family.

The pathogenicity island YAPI^Ye^ of *Y. enterocolitica* strain W22703 contains the homologues *tcaA*, *tcaB*, *tcaC*, and *tccC* and is insecticidal to the tobacco hornworm, *Manduca sexta* (Lepidoptera: Sphingidae), when fed orally; a *tcaA* mutant of the same *Y. enterocolitica* strain lost insecticidal lethality to *M. sexta* [80]. Two *Y. enterocolitica* strains, W22703 and WA314, were shown to have nematocidal activity against *C. elegans* when the strains contained TcaA, though the *E. coli* strain DH5α expressing TcaA clones did not cause pathogenicity in *C. elegans*, implicating additional virulence factors are necessary for full nematicidal activity [81]. The pathogenicity island of *Y. entomophaga*, termed PAI^Ye96^, is composed of insecticidal chitinases and Tc components that cause virulence to *C. zealandica.* Liquid culture supernatants of *Y. entomophaga* were also shown to be insecticidal to the redheaded cockchafer, *Adoryphorus couloni* (Coleoptera: Scarabaeoidae), blackheaded pasture cockchafer, *Acrossidius tasmaniae* (Coleoptera: Scarabaeidae), and *P. xylostella*. In the case of *P. xylostella*, initial apical swelling of gut columnar cells occurred after ingestion of purified Tc from *Y. entomophaga*, followed by complete dissolution of the gut lining [82].

#### 2.2.2. Pseudomonadaceae

*Pseudomonas* spp.: The renowned anti-fungal properties of root-associated bacteria of the genus *Pseudomonas* have been widely used for crop protection and further investigated for viable natural products in both agriculture and medicine. The insecticidal properties (Table 1) of *Pseudomonas entomophila*, *Pseudomonas syringae* and *Pseudomonas fluorescens* were discovered subsequently.

*Pseudomonas fluorescens* insecticidal toxin, or Fit, is an insecticidal proteinous toxin that causes complete loss of turgor pressure and melanization. This toxin has hemocoel-based toxicity in *M. sexta*, and *G. mellonella* [83]. Based on comparisons with *Pseudomonas* strains not expressing the Fit toxin, Fit has been shown to be orally toxic to *S. littoralis*, *H. virescens*, and *P. xylostella. Pseudomonas chlororaphis* (Proteobacteria: Pseudomonadaceae) also expresses a Fit toxin and has oral insecticidal activity [84]. A factor of *P. fluorescens* which correlates to insecticidal activity is the global regulator gene *gacA*. The expression of this gene influences components of the type VI secretion system [85] and *gacA* mutants do not have full insecticidal action [84].

*Pseudomonas entomophila* destroys gut cells of the common fruit fly, *Drosophila melanogaster* (Diptera: Drosophilidae). Based on GC content analysis and the presence of transposases, three prophages, a pyocin-like phage, and a lambdoid phage in the genome, some insecticidal genes of *P. entomophila* were likely acquired through lateral gene exhange. This amount of gene acquisition is less than detected in the genomes of *Pseudomonas putida* (Proteobacteria: Pseudomonadaceae) and *Pseudomonas syringae* (Proteobacteria: Pseudomonadaceae). Although the genome of *P. syringae* does contain *tc* homologs, Tcc and TcdB, similar to those seen in the genome of *P. fluorescens* [86]. Other insecticidal components of *P. fluorescens*, besides the Fit toxin, consist of three TccC toxins, lipases, and exotoxins with hemolytic activity.

## 3. Insecticidal Genes and Toxins Shared by Entomopathogenic Bacteria

The insecticidal components common between different entomopathogenic bacteria often originate from bacteria of widely different life histories. Some bacteria require other organisms such as nematodes or arthropod vectors for their dissemination and introduction to an insect host while others are free-living. This section summarizes the common hypotheses surrounding the issue of why so many insecticidal genes are shared between Gram-positive and Gram-negative bacteria.

### 3.1. Bacillus and Clostridium

#### 3.1.1. A Shared Environment Promotes Gene Transfer

Although Bt is a highly potent insect pathogen in nature, it is rarely associated with diseased insect populations [87]. This is a singular situation for such an effective entomopathogen with specific insect toxins. In this respect, it has been hypothesized that the origin of toxin virulence factors in Bt more likely did not originate through its relationship with insects but through plasmid acquisition [37,88,89]. Interestingly, Bt toxins are reported to be synergistic with other insect pathogens, like *Serratia*, which exists as both a pathogen and symbiont of insects [90]. The ubiquity of Bt where potential insect hosts exist or where insect-associated organisms occur, could have resulted in the acquisition of insecticidal genes.

It is widely accepted that in nature plasmids can be transferred between bacteria [91], however, there is also evidence for gene transfer between organisms of widely different taxa [92,93,94,95,96]. In reference to this, it can be speculated that transfer of insecticidal genes to Bt genome could have occurred when this bacterium occupied the same environment as an insect host. For example, the transfer of plasmid pX016::Tn5401 between mosquitoes and a *Bacillus* species could have occurred when both organisms lived in the same river [97]. Similarly, it is proposed that the virulence factors of *B. anthracis* and the δ-endotoxins genes of Bt, which are on a single plasmid, were recently acquired through horizontal gene transfer [37].

Studies have shown that *Clostridium* spp., *Bacillus* spp., and *P. luminescens* have analogs of the *tc* gene family. In a mass screening of *Bt* strains, 17 out of 81, contained *tccC* gene analogs [98]. Moreover, Mcf toxins are also shared between *Clostridium* spp. and *P. luminescens*. Specifically, the exotoxin from *C. difficile* called cytotoxin B, that causes cytoskeletal reorganization, has a similar mode of action to that of *P. luminescens* toxins Mcf1 and Mcf2. Specifically, the hydrophobic central domain to *C. difficile* toxin B is the portion shared between the Mcf1 and Mcf2 toxins of *P. luminescens*. Both of these toxins are known to have injectable insecticidal activity and have a well characterized cytotoxic motif [51]. Also, the C2 toxins of *C. botulinum* are known to bind actin like Tc toxins. The *tc* genes can be found in various strains of *Bt* including *B. thuringiensis israelensis* bthur0013. In bthur0013 there is an operon with Tc homologs, TccC and TcaCBA, similar to those found in *Photorhabdus* and *Yersinia* spp. [37].

### 3.2. Photorhabdus and Xenorhabdus

#### 3.2.1. Virulence Factors and Specificity in a Mutualistic Relationship

Major similarities exist at the molecular level between *Photorhabdus* and *Xenorhabdus* demonstrated by their insect pathogenic behavior and expression of virulence factors. Secreted proteins and toxins of *Photorhabdus* and *Xenorhabdus* have been shown to play a key role in the dual lifestyle of these bacteria—that is, being avirulent when associated with their nematode hosts and switching to virulence after accessing an insect host. *Photorhabdus* and *Xenorhabdus* secreted proteins play multiple roles during the lifecycle of these bacteria. For example, some are insect-directed virulence factors while others serve as antibiotics to protect the insect cadaver from microbial invaders. Also, chitinases have been found in both *Xenorhabdus* and *Photorhabdus* that have anti-mycotic activity [99]. Two alkaline metalloproteases have been identified from *Photorhabdus*, PrtA and PrtS, both of which inhibit insect antibacterial factors [100]. The secretion of proteins that are both virulent to insects and promote nematode association may be specific to this group of bacteria due to their unique mutualistic relationship.

*Photorhabdus* and *Xenorhabdus* have similar virulence genes and both share a portion of their lifecycle with nematodes. Although their genomes are very similar, strains of *Xenorhabdus* differ from *Photorhabdus* strains by existing as a monoculture in *Steinernema* nematodes whereas *Heterorhabditis* nematodes may contain many different strains of *Photorhabdus* at once [101,102]. It is hypothesized that the differences between the genomes of *Photorhabdus* and *Xenorhabdus* are actually strategies which assist bacteria-nematode association and mutualism.

The *Photorhabdus* genome has higher plasticity with many transposable elements while *Xenorhabdus* lacks this trend; thereby it has limiting genetic variability [101]. The molecular mechanisms that promote plasticity in *Photorhabdus* may explain why so many of its virulence factors have orthologs in other organisms. Other genes which regulate the association of *Photorhabdus* and *Xenorhabdus* with their insect host have been reviewed and explored demonstrating that, although both *Xenorhabdus* and *Photorhabdus* have similar genomes, their strategies to avoid the host immune system, cause pathogenicity, and promote mutualism, differ [103].

The Tc toxins are a superfamily of insecticidal pore-forming toxin proteins that were first identified in *P. luminescens* [104]. The Tc toxins are found in numerous amounts in other organisms and have been further addressed throughout this section. Cloned TcdA, TcdB, and TccC confer oral toxicity [105] and transgenic plants expressing TcdA alone are insecticidal [106]. It has been shown that each subunit of this toxin can have its own insecticidal action [107] and, interestingly, they confer higher toxicity when combined with other Tc subunits [53]. Tc protein subunits are grouped into three categories: “A”, “B”, and “C”. The Tc Bs and Tc Cs are known to make the Tc As more toxic [108].

The Xax proteins are found in *X. nematophila* and *P. luminescens*. The *xax* genes from *X. nematophila* when expressed in *E. coli* have hemocoel-based toxicity acting directly on hemocyte cells. Xax peptides are also found in the *P. luminescens* hemolysin gene designated *plu1962*. The two loci from the genome of *P. luminescens* strain TT01 called *plu1961* and *plu1962* seem to be co-transcribed. This is interesting because the order in which Xax toxins (XaxA and XaxB) are added to cells affect their toxicity [65].

*Xenorhabdus xpt* genes exist on a pathogenicity island like the *tc* genes of *Photorhabdus* and have been demonstrated to confer oral insecticidal activity when cloned into *E. coli* and fed to lepidopterans. *Tc* genes are associated with phage elements and *xpt* genes were likely introduced into their pathogenicity island on transposons [101]. Although not all on a pathogenicity island, Xpt, Tc, Xax, and Sep toxins are similar because different combinations of their clones effects their toxicity when fed to insects [63]. It has been demonstrated that the pathogenicity islands of strains of *Xenorhabdus* are nearly identical*.* This indicates that presence of this pathogenicity island corresponds to an evolutionary advantage or increased fitness. Additionally, it is a hotspot for gene integration or acquisition. There is also evidence supporting the belief that virulence genes were acquired after the original pathogenicity island was integrated into the genome [101].

#### 3.2.2. Novel Targets in the Hemolymph

The genomic organization of the Pir operon of both *P. luminescens* and *asymbiotica* consists of a promoter region, PirA, and PirB; Pir toxin [50,54]. It is unknown how PirA and PirB are differentially expressed when targeting an insect host. Pir toxin is a binary protein that may have an interesting mode of action based upon its homology profile. PirB is homologous to Bt δ-endotoxins in the pore-forming domain and leptinotarsin. Leptinotarsin has been purified from the Colorado potato beetle, *Leptinotarsa decemlineata* (Coleoptera: Chrysomelidae) and has homology to juvenile hormone esterase which is a developmentally regulated protein. Pir toxin does not disrupt insect metamorphosis [54,56,57]. Leptinotarsin is a neurotoxin that stimulates release of acetylcholine at the presynaptic nerve terminal [109,110]. Interestingly, it has been reported that supernatants of *P. luminescens* cultures upregulate insect nervous system genes [111]. The mode of action of Pir toxin and its potential relationship to the insect nervous system is yet to be elucidated.

There are phage-related-loci in both *Serratia* and *Photorhabdus*. In *Serratia* the phage-related loci are pADAP which causes a decreased feeding reaction in infected insects [112]. The phage-related loci of *Photorhabdus* (aka PVC) confers hemolymph-based injectable toxicity on *Galleria* larvae. The target of this virulence factor is hemolymph-circulating phagocytes [113]. Besides the PVC, there are cycle-inhibiting factors (Cif) produced by *P. luminescens.* Although it is unknown how Cif interacts with an insect host, when the c*if* gene is electroporated into *Spodoptera* dervied Sf9 cells they undergo apoptosis and cell cycle arrest [114]. Furthermore, there is a secretion system, termed the type III secretion system or T3SS which is similar to the one characterized in *Yersinia* where effector proteins are translocated through T3SS into host cells [115]. In *Yersinia* the effector protein YopT is a cytotoxic cystein protease whereas the homolog in *P. luminescens*, called LopT, has been shown to prevent phagocytosis [116].

### 3.3. Serratia and Yersinia

#### 3.3.1. Presence of Insecticidal Genes

Both *Yersinia* and *Serratia* share Tc homologs. Analysis of the *sep* genes from *Serratia* showed that they are a cluster associated with pADAP and are a part of a horizontally mobile region*.* The *sep* insecticidal toxin genes from *S. entomophila* and *S. proteomaculans* occur in *Y. frederiksenii.* The *Y. frederiksenii sep*-like genes were termed *tcYF1* and *tcYF2* and have correlating high percent identity to *sepA* and *sepB* genes [76]. In *Y. pestis*, toxin complexes A, B, and C (TcaA, TcaB, and TcaC) are termed *yitA*-*C*. There are insecticidal regions of homology between *P. luminescens*, *Y. pestis*, and *Salmonella* genes termed *tcdB1*, *yitC*, and *spvB*, respectively. The *Salmonella* gene *spvB* is an effector of the T3SS secretion system [117]. Different *Yersinia* species have a common genomic backbone. When analyzing the presence of insecticidal genes between all *Yersinia* species the *tc* operons have been inserted into a common region of the greater *Yersinia* backbone [118].

#### 3.3.2. The Presence of Insecticidal Toxins May Increase Pathogenicity and Infectivity

The discovery of insecticidal components produced by *Y. pestis* is interesting because these bacteria require insect vectors (such as fleas) for human infectivity. Studies conducted by Spinner *et al*. 2012 have shown that two Tc proteins of this bacterium, YitA (TcaA-like) and YipA (TccC-like), are highly expressed when the bacteria are inside the flea. However, it has been shown that deletion of these two toxins does not prevent flea infection [119]. The presence of the *tc* genes in *Yersinia* spp. suggests that insects are a natural host of these bacteria. Studies have shown that different *Yersinia* strains tested for oral toxicity against *M. sexta* cause higher mortality when the strains have the toxin complex pathogenicity island; however, their insecticidal activity showed no difference when they were subcutaneously injected into *G. mellonella* larvae [78].

The high similarity of Tc proteins in various strains of *Y. pestis* suggest that they may have similar roles in infectivity of fleas compared to *Y. psuedotuberculosis* where Tc proteins are less conserved [120]. However, Tcs of *Yersinia* spp., which are pathogenic to humans (*Y. pestis*, *Y. pseudotuberculosis*, and *Y. enterocolitica*), seem to play a differential role in mammal infectivity. There is differential specificity between the Tcs obtained from *Y. pseudotuberculosis* and *Y. pestis* on human epithelial cells. The TcaA from *Y. pseudotuberculosis* caused human-derived enterocytes to display actin ruffles and increased vacuolization. The TcaA from *Y. pestis* causes multinuclei formation. The TcaA from *P. luminescens* does not affect the actin of mouse-derived cells [53]. This level of specificity suggests TcaC of the insect toxin complex assists in human pathogenicity [121]. In *Y. enterocolitica*, an intestinal pathogen to humans, *tc* genes have been shown to assist in gut colonization of mice and have higher prevalence in more virulent strains of *Y. enterocolitica* [77].

The toxins of different entomopathogens may assist increased pathogenicity to target insect populations. Jeong *et al*. showed that *S. marcescens* produces insecticidal oral toxins [122]. Interestingly, there is a synergistic insecticidal effect between *S. marcescens* chitinases [123] and Bt Cry1Ac toxin [90]. There are also synergistic effects between Bt Cry1C and the supernatant of *S. marcescens* to tobacco cutworm, *Spodoptera litura* (Lepidoptera: Noctuidae), but not cabbage moth, *Mamestra brassicae* (Lepidoptera: Noctuidae), *P. xylostella*, or the smaller tea tortrix, *Adoxophyes honmai* (Lepidoptera: Tortricidae) [124]. The insecticidal genes of Bt and *Serratia* may be able to work in synergism with each other in nature.

### 3.4. Pseudomonas

*Pseudomonas fluorescens* has both insecticidal and anti-mycotic properties. The virulence factor, Fit protein toxin, identified in *P. fluorescens* has been shown to have insecticidal properties and based upon homology, the Mcf proteins identified from *P. fluorescens* are suspected to have similar modes of action to that of Fit (See Section 2.2.1). When Fit or Mcf are ingested by lepidopteran larvae the toxin causes complete loss of turgor pressure and the characteristic “floppy” phenotype [83,84].

Another *Pseudomonas* species, *P. entomophila*, lacks the characteristic T3SS which most insecticidal bacteria possess to inject effector molecules into their host [125]. The virulence factors associated with *P. entomophila* have been shown to cause death of gut cells, suggesting an oral route for toxicity [126]. Many other insecticidal virulence factors have been identified in *P. entomophila*, some of which are the characteristic Tc analogs associated with a gene acquisition event [86].

## 4. Neurobiology of Hemolymph-Based Toxicity

This section summarizes the current knowledge of hemolymph-based toxicity caused by various entomopathogenic bacteria in relation to neurobiology of insect hosts. We also discuss potential application of this knowledge for use in the management of lepidopteran pests.

### 4.1. Neurotoxins and Their Application in Pest Management

Synthetic pesticides are the most widely accepted strategies for insect pest management. Most of the synthetic pesticides, such as organophosphates, pyrethroids, and phenylpyrazoles, target the nervous system of insects. When synthetic pesticides are applied as foliar sprays or used in urban settings they have the potential to persist in the environment with deleterious effects [127,128]. Furthermore, these insecticides lack pest specificity and promote development of pest resistance [129,130,131]. Synthetic pesticide resistance leads to a problem often referred to as the pesticide treadmill, meaning that the more often a pesticide is used, the more potent and highly concentrated a pesticide must be for efficacious future use [132].

The lack of specificity of synthetic pesticides results in similar toxic effects between insects, humans [133] and on other non-target organisms [134]. One example is the synthetic pesticide ingredient amitraz. This insecticide is an acaracide and an octopamine receptor agonist of the nervous system of invertebrates [135]. In the United States, it has been most commonly used to control ticks and fleas on dogs, mites on cotton, and insect pests on pear trees [136]. Amitraz also stimulates the α2-adrenoceptors of the human nervous system [137]. Some natural products used in organic agriculture have modes of action similar to synthetic pesticides despite their natural origins. For example, pyrethrum from chrysanthemums, *Tanacetum cinerariifolium* (Asterales: Asteraceae), have pyrethrins, chrysanthemates and pyrethrates with neurotoxic action similar to that of synthetic pyrethroids [138]. Another example is spinosyns from soil actinomycetes which disrupt acetyocholine neurotransmission [138]. One organic agricultural product with insect specificity is the azadirachtins class of organic chemicals, which is derived from the Indian neem tree, *Azadirachta indica* (Sapindales: Meliaceae), which block insect molting hormones thereby disrupting insect development [138].

One way to circumvent the damaging non-target effects of synthetic pesticides, which share common modes of action between humans and insects, is the consideration of naturally occurring entomopathogens and/or the toxins they produce [139]. For example, Bt foliar sprays are organic, have high specificity, and have negligible environmental impact compared to their synthetic counterparts [140]. Another alternative to synthetic pesticides is the use of toxins produced by mites, spiders, and other venomous organisms which also have been shown to have neurotoxic effects on insects [141]. Various studies have shown that some of the genes encoding these toxins can be expressed in transgenic plants and contribute to a decrease of the target insect population [142].

As mentioned, many neurotoxins have been discovered from invertebrates. For example, leptinotarsin, isolated and identified from the Colorado potato beetle, has been shown to disrupt the release of acetylcholine at the presynaptic nerve terminal of rat synaptosomes [109,110]. Although peptides of leptinotarsin have shown homology to both juvenile hormone esterase (JHE) of insects and Cry toxin of Bt, there is no evidence that leptinotarsin has JHE activity. Because of the way proteinous neurotoxins interact and disrupt neural tissue function, they can be used to study the physiological consequences of the nervous system dysfunction [143]. Understanding their mode of action and interaction with the insect nervous system can make them a powerful tool with application in insect pest management.

#### 4.1.1. Insect Paralysis Caused by Bt

Once Bt Cry toxins are ingested by lepidopteran larvae a number of effects concurrent to toxicity occur including paralysis. Paralysis is such a predominant characteristic that early studies reported paralytic effects caused by Bt as a preliminary way to discriminate among different insecticidal *Bacillus* species and strains [144]. Paralysis induced by Bt can be categorized by insect type. Type I insects are described by the symptom of whole body paralysis. The larvae have been described as becoming inactive causing them to fall off their host plant (Table 2). This has been demonstrated to be caused by increased pH in the hemolymph. In type II insects, paralysis symptoms are limited to gut movement. Paralysis is thought to be caused by the breakdown of the epithelial integument, thereby inhibiting function and movement [145]. Gut paralysis in Type II insects has been described as cessation of feeding, frass production, or microscopic evidence of muscles surrounding the gut relaxing (Table 2).

Very few studies have discussed the potential role of the insect nervous system, brain, or neuromuscular junction in insect gut or whole body paralysis. Peristalsis in Lepidoptera is controlled by the stomatogastric nervous system [146]. The frontal ganglion controls the ability of the foregut to empty food into the midgut. Peristalsis is controlled by the frontal connectives so if digestion has ceased then this aspect of the nervous system may have been targeted. However, larva regurgitation is a common defense response during ingestion of plant defense molecules. Midgut paralysis, the cessation of muscles emptying food from the foregut to the midgut, causing regurgitation, is not usually distinguished from regurgitation as a defense response. The toxins of insect pathogens which have both oral and injectable toxicity could selectively silence specific insect nervous tissues surrounding digestion and midgut muscle movement. Understanding toxins of this nature would help determine the function of these tissues in insect behavior, especially regarding food consumption.

One Cry toxin of Bt, Cry1C, targets the nervous system [28] and the gut [147] of various Lepidoptera species. Although Cry toxins are orally toxic compounds acting as gut poisons, upon observing a range of gut paralytic effects the role of the nervous system has been discussed [148]. Some of the neurotoxic effects of Cry toxins may be explained by their homology to both Pir toxin from *P. luminescens* and neurotoxin from *L. decemlineata* [56].

#### 4.1.2. Neurotoxicity in Other Bt Strains

Neurotoxic symptoms have been observed when Bt var. *israelensis* (Bti) are directly injected into *T. ni*. Higher doses of Bti stops heart activity when injected into the hemolymph. Moreover, a loss of motor activity, paralysis and flaccidity was observed. Similar but more benign symptoms occurred when Bti was injected into mice. Oral feeding of Bt *kurstaki* strain to *T. ni* resulted in vomiting while injection had no adverse neuromuscular affects. The proteins conferring neurotoxicity were components of the crystal endotoxin from Bti [149].

The neurotoxic effects of Bti were further explored in the American cockroach, *Periplaneta americana* (Blattodea: Blattidae). The presynaptic nerve terminal function was suspected to be blocked whereas the postsynaptic membranes and axons in the ventral nerve cord remained unaffected. The sixth abdominal ganglion transmitter release, calcium uptake, and complete blockage of transmitters were observed. Rat muscle cultures treated with a purified crystal protein from Bti resulted in degeneration. The mode of action described was related to Na^+^/K^+^-ATPase damage upon incubation and K^+^ levels decrease while Na^+^ levels increase within muscles cells with increasing Ca^2+^ influx [150].

**Table 2:** Paralytic effects of ingested Bt on various lepidopteran families and species.

Family	Species	Bt component	Response
Noctuidae	*Spodoptera* spp.	Not specified	No paralysis
	*H. virescens*	Bt var. *kurstaki*	Midgut paralysis; Intermittent whole body paralysis with recovery to body paralysis
	*T. ni*	Not specified	Type I paralysis
Saturniidae	*Philosamia ricini*	Bt var. *sotto* crystals	Whole body paralysis, Type I
Crambidae	*Ostrinia nubilalis*	Bt var. *thuringiensis* crystalline paraspores	Gut paralysis
Pyralidae	Not specified	Cry proteins	Paralysis
	*G. mellonella*	Spores and crystals derived from Thuricide	No paralysis, Type III most susceptible to spores
	*Ephestia cautella*	Not specified	Type II paralysis
Sphingidae	*Phlegathontius quinquemaculatus*	Thuricide (International Minerals and Chemical Corp., Libertyville, IL, USA)	Abnormally quiescent, cessation of feeding and slow death, no paralytic effect which was directly compared to synthetic insecticide induce paralysis
Erebidae	*L. dispar*	Not specified	Type II paralysis
Plutellidae	*P. xylostella*	Bt biological products	Decreased movement with subsequent paralysis
	*P. xylostella*	Bt var. *kurstaki*-HD1, sprayed bacterial suspensions	Reduction of movements to stoppage, limp, loss of agility and movements slow, unresponsive to touch
Papilionidae	*Papilio demoleus*	Bti Berliner spore	Fairly rapid paralysis followed by an increase of blood alkalinity after ingestion of spores
Gelechiidae	*Pectinophora gossypella*	Bt δ-endotoxin	Evidence of gut paralysis by histological investigation where gut muscles surrounding the disorganized epithelium are relaxed
Bombycidae	*B. mori*	*Bacillus sotto*	Paralysis within four hours
	*B. mori*	Bt var. *sotto*	Paralysis
	*B. mori*	Not specified	Type I paralysis
	*Protoparce quinquemaculata*	Bt	General paralysis
	*P. quinquemaculata*	Not specified	Type I paralysis
	*Protoparce sexta*	Bt crystals	General paralysis
	*Antheraea pernyi*	Bt crystals	General paralysis
Pieridae	*Colias eurytheme*	Bt var. *thuringiensis*	No paralysis
	*Pieris rapae*	Not specified	Type II paralysis
Hesperiidae	*Urbanus acawoios*	Bt var. *kurstaki* wettable powder	Decreased movement after 10 h, classified as likely Type II because no general paralysis
Tortricidae	*Choristoneura fumiferana*	Bt Dipel foliar spray	Interruption of feeding due to gut paralysis resulted in reducing rate of development

## 5. Areas for Future Study

The recent discoveries of numerous insect toxin analogs have raised questions about the origin of virulence factor genes of entomopathogenic bacteria. This is because it is those genes which define the very nature of entomopathogens and their pathogenic relationships with their target hosts. There are also symptoms which occur in entomopathogenicity which have no defined virulence factor associated, raising the question of who is the culprit. This section addresses some of these questions and briefly summarizes the work that has been done to raise these questions.

### 5.1. Why Are There so Many Insecticidal Genes in Bacteria?

One potential answer to the question of why there are so many insecticidal genes lies in the nature of Lepidopteran larvae behavior. Many larval stages spend most of their time ingesting food and bacteria along with it. Bacteria exist in habitats where these insects reside such as soil, phylloplane, or in an endophytic stage. Therefore, it is no surprise that potent virulence factors of entomopathogenic bacteria have an oral route for toxicity, like the Tc superfamily and Cry toxins.

The gut of lepidopteran larvae is also a reservoir of diverse bacterial populations that interact with one another, competing for both space and nutrients. It is known that organisms in constant competition for resources are in an atmosphere which fosters adaptation [151]. For example, *P. luminescens* has adapted to outcompete surrounding microbes by secreting antibiotics. As a result, after infecting an insect host, *P. luminescens* dominates the insect cadaver [152].

Previous to molecular genome comparisons, bacteria were categorized by their specific pathogenic effect. For example species within the *B. cereus* sensu lato are distinguished by the diseases they cause in humans and insects. Now we come to find through bioinformatic analysis that similar insecticidal toxins have been identified in their genomes. The high degree of similarity between genomes of different *Bacillus* spp. and the identification of common insecticidal genes is probably due to plasmid shuffling, horizontal gene transfer events, and phage elements surrounding the toxin genes. It can be hypothesized that these similarities could be explained by their shared environment, including a common host, which promoted the exchange of genetic material. For example, recent genome analysis of *Yersinia* and *Serratia* have shown evidence of similar plasmid shuffling events in *Y. pseudotuberculosis* [153] and transposable elements in *S. entomophila* [154].

As more bacterial genomes are sequenced, more virulence factors will be identified between different taxa. So far, analogs within the *tc* gene superfamily, originally identified in *P. luminescens*, have also been identified in *Yersinia*, *Xenorabdus*, *Bacillus*, *Clostridium*, *Salmonella* and *Serratia* [155] potentially with more to come. *Photorhabdus* Pir proteins have homology to Bt Cry toxins and Mcf toxins have analogs in both *Clostridium* and *Pseudomonas* [56].

### 5.2. Why Nematode-Vectored Entomopathogenic Bacteria that Are Directly Delivered to the Host’s Hemocoel Produce Oral Toxins?

*Photorhabdus luminescens* toxins have both oral and hemolymph-based toxic capability. Hemolymph-based toxicity from an insect pathogen which can be delivered directly into the insect makes sense but the orally toxic nature of specific and highly pathogenic toxins is curious. The *tc* genes are found in many organisms which are not directly delivered into the hemolymph. The origin of the *tc* genes could be better understood by investigating the ancestry of bacteria with Tc toxins and the origins of nematode association with bacteria.

### 5.3. Why Is the Mechanism of Gut Paralysis Limited to Bt?

As mentioned above, Bt toxins are known to cause gut and whole body paralysis. It has been demonstrated that Bt causes whole body paralysis by the release of K^+^ from the compromised midgut into the hemolymph or from intracellular sources such as K^+^ channels, in the absence of midgut K^+^ [156]. Paralysis effects can be mimicked, by injecting alkaline buffers directly into the hemocoel [145]. Most neurotoxins, in particular those derived from insect hunting arthropods (*i.e.*, spiders), act on major ion channels to produce their paralyzing effect [157]. Bt has been previously reported to disrupt ion channels in the midgut [158,159] and ion channel formation at gut neuromuscular junctions could be one way Bt causes paralysis. The binding of Bt to ATP binding cassettes also has the potential to disrupt ion transport across a membrane. ATP binding cassettes utilize ATP to transport various substrates across membranes and have recently been identified as Bt receptors. Insects harboring mutant ATP binding cassettes in their genome are resistant to Bt. This may be another mechanism to investigate regarding Bt gut paralysis.

## 6. Conclusions

Without any doubt, Bt, whether incorporated into a foliar spray or toxins expressed in transgenic plants, is regarded as the premier entomopathogen used in insect pest management. Recently, similar toxins to that of Bt have been identified throughout the bacterial kingdom. These finding may have two types of implications: (1) the development of novel tools for insect pest management and (2) the gain of further knowledge of the origin of entomopathogens and their associated virulence factors. For example, it would be interesting to investigate if the combination of virulence factors of two different entomopathogenic bacteria such as *Photorhabdus* and *Bacillus* delay resistance. Combining toxins with different modes of action may delay the onset of resistance by forcing insects to develop two separate mechanisms of resistance. Moreover, their combination could result in a more lethal insecticide that would result in a better control tactic for a problematic insect pest.

Furthermore, the delivery mechanism of *Photorhabdus*, *i.e.*, directly released by nematodes into the insect’s hemocoel, suggests the existence of virulence factors with novel tissue targets in the Lepidoptera that can be further investigated. If proven successful the combination of newly identified hemolymph-based toxins with conventional orally-based toxins would expand their application in insect pest management.

Entomopathogens in general have a history of horizontal gene transfer events shuffling toxin-containing plasmids and pathogenicity islands between each other. The acquisition of insecticidal genes may be a strategy that developed to onset virulence once bacteria were ingested, thereby, broadening the availability of possible nutrient sources. Recognizing that insect virulence factor genes are present throughout the bacterial kingdom can broaden our knowledge of the nature of entomopathogenicity and the role of bacterial symbionts as entomopathogenic partners.
